# Supplementary material for: Why Do Women Not Use the Bathroom? Women’s Attitudes and Beliefs on Using Public Restrooms
Source: Int J Environ Res Public Health. 2020 Mar 20;17(6):2053. doi: 10.3390/ijerph17062053 (PMC7142493; doi:10.3390/ijerph17062053)
Supplement: Supplementary file 1 [file ijerph-17-02053-s001.pdf]

International Journal of Environmental Research and Public Health

MDPI

Supplementary Material

Table 1. The coding system developed for analyses of open-ended responses.

Code	Category	Description	Examples
**1**	**Restroom type**	**Discussion pertains to work/school/public restrooms**	
1.1	Work/school	Response pertains to work and school restrooms	Included in every quote in "Restroom Type" column
1.2	Public	Response pertains to public restrooms	Included in every quote in "Restroom Type" column
**2**	**Quality**	**Discussion centers on quality of bathroom as a factor that contributes to limited bathroom use**	
2.1	Cleanliness/tidiness	Discusses cleanliness, tidiness, and neatness of bathrooms	E.g., Trash on the floor, wet floor, hair in the sink, and overflow of trash can. Also, code for the words "clean" or "dirty" in quotes generally speaking. If it is not, generally speaking, see rule 2.3 below.
2.2	Amenities	Discusses presence/quality of amenities in bathrooms	E.g., Paper towels, broken equipment, no soap, and older facility
2.3	Sanitation/hygiene	Discusses sanitation, hygiene, and sterility of bathrooms	Only use this code for terms such as hygiene, germs, sanitation, blood on seat, pee on seat, not flush, clogged toilet, and filthy. Also use this code if coed bathroom is used, unless the issue is safety-related only. Additionally, use this code when "dirty" is coupled with a specific reason for not using the bathroom
2.4	Smell	Discusses odors of bathrooms	E.g., Smell of bowel movement and air freshener
2.5	Other	Other discussion on quality of bathrooms that does not fit in the above categories	General quality quotes
**3**	**Psychological factors**	**Discussion centers on psychological factors that contribute to limited bathroom use**	
3.1	Safety-related concerns	Discusses safety-related concerns associated with bathroom use	E.g., Prior trauma
3.2	Human interaction	Discusses types of human interactions associated with bathroom use	E.g., Do not want to run into people and social awkwardness. Also code if participant discusses that she does not want to inconvenience others
3.3	Contamination fear	Discusses obsessions related to bathroom use	E.g., I obsess about germs, I have OCD, I am afraid of infection, or I am afraid of germs. Use this when a fear code is combined with a germ

			code or cleanliness code.
3.4	Emotion	Participant discusses emotions related to bathroom use	
3.4.1	Fear/stress/worry	Discussion centers on fear, stress, worry, and/or anxiety	
3.4.2	Embarrassment	Discussion centers on embarrassment or self-consciousness	
3.4.3	Disgust	Discussion centers on disgust, grossness, “ickiness”, and/or nastiness	E.g., Expresses directly that bathroom is gross or icky. Does not cooccur with 2.3
3.5	Other psychological factor	Other discussion on psychological factors that does not fit in the above categories	
**4**	**Accessibility**	**Availability of accessible bathroom**	
4.1	Distance/findable	Discusses distance and difficulty finding a bathroom as a deterrent to bathroom use	E.g., Distance of restroom and difficulty finding a restroom
